# Tau: The Center of a Signaling Nexus in Alzheimer's Disease

**DOI:** 10.3389/fnins.2016.00031

**Published:** 2016-02-09

**Authors:** Shahzad S. Khan, George S. Bloom

**Affiliations:** Department of Biology, University of VirginiaCharlottesville, VA, USA

**Keywords:** Alzheimer's disease (AD), Amyloid-β oligomers (AβOs), neurofibrillary tangles (NFTs), tau phosphorylation, synaptic dysfunction

## Abstract

Tau is a microtubule-associated protein whose misfolding, hyper-phosphorylation, loss of normal function and toxic gain of function are linked to several neurodegenerative disorders, including Alzheimer's disease (AD). This review discusses the role of tau in amyloid-β (Aβ) induced toxicity in AD. The consequences of tau dysfunction, starting from the axon and concluding at somadendritic compartments, will be highlighted.

## Introduction

Alzheimer's disease (AD) is a neurodegenerative disorder that causes an insidious decline in cognitive function. The greatest risk factor for AD is age and the chances of developing the disease increases two-fold every 5 years after age 65. As the population continues to live longer, the toll that AD inflicts on healthcare costs for affected individuals will continue to rise. Thus, there is an urgent need to improve our understanding and therapeutic treatment of AD.

One of the barriers preventing the expeditious treatment of AD is our inability to detect at-risk individuals early enough for effective intervention. A diagnosis of AD can only be confirmed at autopsy following the detection of extracellular plaques containing Aβ peptides and intracellular neurofibrillary tangles composed of the neuron-enriched, microtubule-associated protein (MAP), tau. Since mutations in the amyloid precursor protein (APP) gene are linked to AD onset, and plaques often precede the formation of tau tangles, the amyloid cascade hypothesis, which states that Aβ initiates hyper-phosphorylation and aggregation of tau, and overall AD pathogenesis, was developed (Hardy and Higgins, 1992).

Although, it has been popular to focus primarily on Aβ in AD, tau plays an equally important role in AD pathogenesis. For instance, individuals with substantial plaque loads but no evident tau pathology can lead healthy lives with no symptoms of cognitive decline (Sperling et al., 2009). Additionally, research has long supported the notion that tau is the major component of neurofibrillary tangles that positively and robustly correlate with AD severity Grundke-Iqbal et al., 1986; Nukina and Ihara, 1986; Kondo et al., 1988; Kosik et al., 1988; Braak and Braak, 1991; Götz et al., 2001; Lewis et al., 2001). Tau depletion also protects against Aβ-associated neuron death (Leroy et al., 2012; Nussbaum et al., 2012). Thus, it is generally accepted that tau dysfunction, manifested as hyper-phosphorylation and aggregation, are major proximal causes of neuron loss in AD (Bloom, 2014). This review emphasizes the role of tau as a central player in a pathogenic signaling nexus that underlies AD.

## Background on tau

Tau was originally identified as a predominant MAP present in mammalian brain (Weingarten et al., 1975). In the CNS, alternative splicing leads to the formation of six isoforms (Goedert et al., 1989a). Variation among the six isoforms lies in the number of exons expressed at the N-terminus (0, 1, or 2) and microtubule binding repeat domains near the C-terminal end of the protein (3 or 4). During early stages of mammalian development, the 3 repeat domain tau isoforms predominate and it is heavily phosphorylated (Goedert et al., 1989b). A proposed function for elevated tau phosphorlyation during development is that it contributes to synaptic plasticity (Frandemiche et al., 2014). As the brain ages, however, phosphorylation of tau decreases and the presence of 4 repeat to 3 repeat tau reaches an approximate 1:1 ratio (Goedert et al., 1989a,b; Himmler et al., 1989; Kosik et al., 1989; Goedert and Jakes, 1990; Hong et al., 1998).

## Tau function

The first antibody generated against tau helped determine that its expression is predominantly axonal (Binder et al., 1985). Subsequent experiments using the same antibody identified tau, and what would be later shown as hyper-phosphorylated tau, as the primary component of neurofibrillary tangles (Grundke-Iqbal et al., 1986; Wood et al., 1986; Kondo et al., 1988; Kosik et al., 1988). The putative functions of tau include stimulation of tubulin polymerization, stabilization of microtubules (Witman et al., 1976), and a “speed bump” property whereby tau constrains the fast transport of organelles along microtubules (Stamer et al., 2002; Dixit et al., 2008). Tau is most concentrated in the distal portions of axons, where it helps regulate microtubule dynamics.

Methods designed to knockdown or knockout (KO) tau have identified additional roles for the protein in the CNS. Early work on tau depletion using antisense nucleotides suggested that tau is required for proper axon development and neuronal polarity (Caceres and Kosik, 1990). However, follow-up studies *in vivo* using tau KO mice were less convincing (Harada et al., 1994), and subsequent work in several tau KO strains reported no change in reproduction, physical appearance, or behavior (Dawson et al., 2001; Tucker et al., 2001). Nevertheless, during mouse postnatal development reduction of tau alters the migration and morphology of neurons, and also intracellular mitochondrial transport (Sapir et al., 2012). In *Drosophila*, global tau KO is developmentally lethal and targeted KO in neurons or the eye results in progressive neurodegeneration (Bolkan and Kretzschmar, 2014). However the lethality and neurotoxicity of tau KO in *Drosophila* might reflect a general paucity of microtubule-associated proteins in flies.

Much of the original work using tau KO mice extends to 6 months of age, leaving the long-term effects of tau depletion uncertain. Recent work has attempted to investigate the effects of prolonged tau ablation, with very inconsistent results. Work from one group demonstrated that aged tau KO mice (older than 6 months) develop iron accumulation, motor deficits, Parkinsonism with dementia, significant brain atrophy, and impaired Y-maze performance (Lei et al., 2012, 2014). Similarly, others have reported motor deficits in aged tau KO mice (Ma et al., 2014; Lopes et al., 2016), or decreased brain weight and mild hyperactivity in aged tau KO homozygotes (Li et al., 2014). Evidence of impaired learning, as determined by Barnes maze performance, was also recently reported (Regan et al., 2015).

However, other studies reported no difference in iron accumulation (Li et al., 2014), parkinsonian abnormalities in dopamine levels (Li et al., 2014), dopamine-related motor deficits (Morris et al., 2013; Ahmed et al., 2014; Li et al., 2014), or impaired Y-maze performance in aged tau KO mice (Li et al., 2014). Furthermore, results demonstrated that Morris water maze performance is either improved or unaffected (Morris et al., 2013; Ahmed et al., 2014) which presents conflict to other data (Ma et al., 2014). Likewise, the literature reports inconsistent fear conditioning results in aged tau KOs. One study showed no difference in contextual fear conditioning (Li et al., 2014) while another reported impaired cue and contextual fear conditioning (Ahmed et al., 2014). The literature also reports contrasting data on long-term depression (LTD), with severe impairments in long-term potentiation and no effect on LTD shown by one group (Ahmed et al., 2014), and deficits in LTD demonstrated by others (Kimura et al., 2013; Regan et al., 2015).

These discrepancies might reflect strain-dependent phenotypic differences among the various tau KO mouse lines. Hence, as more studies are completed, and as methods become more standardized, we will be better able to resolve what consequences, or lack thereof, arise with prolonged tau ablation. Understanding the effects of prolonged tau ablation is not only important to elucidate tau function, but is also necessary to ascertain the best therapeutic strategy to employ when treating tau-related neurodegenerative disorders.

## Tau impairment at the axon in AD

Among tau post-translational modifications its phosphorylation is the best characterized. There are 80 serine or threonine and 5 tyrosine sites at which tau can theoretically be phosphorylated. Once tau becomes aberrantly phosphorylated its functional capacity to stabilize microtubules is reduced, contributing to axon deficits in AD. Furthermore, axonal swellings, or varicosities, that are frequently observed during early-stage AD, are hypothesized to reflect tau-associated defects in transporting cargo-containing vesicles (Krstic and Knuesel, 2012**)**. Expression of tau phosphomimics lends supporting data to this theory by demonstrating that sustained tau (psuedo)phosphorylation impairs its axonal transport and degradation (Rodríguez-Martín et al., 2013). Other tau post-translational modifications, such as *cis* or *trans* isomerization, also affect the ability of tau to maintain microtubule assembly at axons (Nakamura et al., 2012).

In support of the amyloid cascade hypothesis, Aβ oligomers (AβOs) are reported to impair the ability for tau to stabilize microtubules. Our lab previously reported that pre-fibrillar Aβ stimulates tau-dependent disassembly of microtubules (King et al., 2006**)**. It was likewise reported that Aβ treatement promoted tau hyperphosphorlyation, microtubule-related deficits, and organelle dysfunction (Silva et al., 2011). Similarly, tau is implicated to trafficking deficits in cell surface receptors, particularly those that bind glutamate, following AβO treatment (Li et al., 2009; Hoover et al., 2010). Thus, under atypical conditions Aβ and tau interact to cause significant microtubule and transport dysfunction.

## Somadendritic tau

A conspicuous property of tau in AD is its ectopic mislocalization to somatodendritic compartments (Götz et al., 1995; Hoover et al., 2010). It is hypothesized that an Aβ and tau interaction causes the synaptotoxicity commonly observed in AD. For instance, double transgenic mice that overexpress the human forms of APP (containing the Swedish and London mutations, for example) and WT tau acquire significant dendritic spine loss with age (Chabrier et al., 2012, 2014). Mechanistically, Aβ exposure promotes complex formation between the non-receptor tyrosine kinase, fyn, and PSD95, in a tau-dependent manner, to mediate aberrant activation of the NMDA receptor in dendritic spines (Ittner et al., 2010). Some synapse dysfunction following Aβ exposure also requires formation of a complex containing fyn and the cellular prion protein, and fyn-dependent phosphorylation of tau (Larson et al., 2012). Tau phosphorylation by other kinases, such as AMP-activated kinase (AMPK) is further necessary for AβO synaptotoxicity (Mairet-Coello et al., 2013). In addition to the requirement of tau for the AβO-initiated activation of the NMDA receptor, recent work has shown that AβOs elicit phospho-tau infiltration to dendrites and AMPA receptor dysfunction (Miller et al., 2014).

Importantly, cell-cycle re-entry (CCR) by post-mitotic neurons preludes much of the massive neuron death that occurs in AD (Arendt, 2012) and is also associated with impaired synaptic plasticity (Arendt and Brückner, 2007). AβO treatment of primary neurons results in activation of the kinases CaMKII, PKA, and fyn, which induce ectopic CCR by a mechanism that relies on site-specific tau phosphorylation catalyzed by those kinases (Seward et al., 2013). Neuronal CCR is also present in hAPPJ20 AD model mice at 6 months, but absent in comparable tau KO littermates (Seward et al., 2013).

## Nuclear tau

Although, it is predominately expressed in the axon, tau can also be found in other cellular compartments, including the nucleus, under normal physiological conditions. Its nuclear role is unresolved, but one theory proposes a protective role against DNA damage for nuclear tau, depending on its phosphorylation state (Sultan et al., 2011). Interestingly, tau was recently shown to induce chromatin relaxation, which subsequently leads to DNA damage and global changes in transcription (Frost et al., 2014).

## Conclusions

The current understanding of tau identifies its hyper-phosphorylation and subsequent mislocalization as seminal steps for AD pathogenesis. Once it becomes appropriately phosphorylated, tau loses its affinity for microtubules and becomes potently cytotoxic (Alonso et al., 2010). Over the course of AD, hyper-phosphorylated tau ectopically enters the somadendritic compartment, where in conjunction with AβOs, it promotes excitotoxicity at synapses. Additionally, tau phosphorylation modulates DNA integrity under cellular stress, and global changes in protein transcripts. Inhibiting aberrant tau phosphorylation may prove useful in the treatment of AD. However, targeting tau phosphorylation will require a greater understanding on how site-specific tau phosphorylation alters its function.

## Author contributions

SK wrote an advanced draft of this mini-review, which was then edited by GB.

### Conflict of interest statement

The authors declare that the research was conducted in the absence of any commercial or financial relationships that could be construed as a potential conflict of interest.
